# Comparative Study on the Chemical Components and Gastrointestinal Function on Rats of the Raw Product and Licorice-Simmered Product of *Polygala tenuifolia*

**DOI:** 10.1155/2021/8855536

**Published:** 2021-01-07

**Authors:** Yueli Cui, Xin Zhao, Yuqiu Tang, Yanxue Zhang, Le Sun, Xuelan Zhang

**Affiliations:** ^1^School of Pharmacy, Shandong University of Traditional Chinese Medicine, Jinan 250355, China; ^2^Jinan Central Hospital of Shandong Province, Jinan 250013, Shandong, China; ^3^Shandong Provincial Collaborative Innovation Center for Quality Control and Construction of the Whole Industrial Chain of Traditional Chinese Medicine, 4655 Daxue Road, Jinan 250355, Shandong, China

## Abstract

The root of *Polygala tenuifolia* Willd. (Polygalaceae) (PT) has been listed as a nootropic, anti-inflammatory, and antipsychotic medicine that can cure insomnia. Raw PT (RPT) is toxic and must be processed before clinical use. Licorice-simmered PT (LPT) is one of the most common processed products. We conducted this study in order to investigate the differences in chemical components and gastrointestinal function between RPT and LPT. We used principal component analysis (PCA) and quantitative analysis to study the differences in the chemical components. Animal experiments were conducted to evaluate the effects of PT on the gastrointestinal function of rats before and after simmering. Pathological sections of gastrointestinal tissues, serum hormone levels, and inflammatory cytokines were observed. The PCA results demonstrated that obvious separation was achieved between the RPT and LPT samples. Tenuifoliside B (TFSB), 3,6'-disinapoyl sucrose (DSS), tenuifoliose A (TFOA), tenuifoliose H (TFOH), onjisaponin B (OJB), onjisaponin Z (OJZ), and total saponins levels were decreased after licorice processing, while glomeratose A (GA) and 3,4,5-trimethoxycinnamic acid (TMCA) levels were markedly increased. Compared to the control group, the RPT groups exhibited dramatically lower levels of gastrin (GAS), motilin (MTL), and substance P (SP) and markedly higher levels of vasoactive intestinal peptide (VIP) and somatostatin (SS), but the LPT groups exhibited no significant differences in the above indexes. The levels of interleukin-6 (IL-6), interleukin-8 (IL-8), and tumor necrosis factor-*α* (TNF-*α*) in gastrointestinal tissue were markedly increased in the low RPT (L-RPT), high RPT (H-RPT), and H-LPT groups, showing a certain inflammatory effect, but the inflammatory effect in the L-LPT group was relatively weak. Licorice simmering can effectively reduce the inhibitory effect of RPT on gastrointestinal function in rats and reduce damage to gastrointestinal tissue. This study provides a scientific basis for research on the processing mechanism and clinical application of PT.

## 1. Introduction

Chinese medicine processing techniques are characteristics of traditional Chinese medicine (TCM). Raw Chinese medicines are often processed before clinical application, which is called “paozhi” in Chinese, to enhance their efficacy, reduce their toxicity, change their properties, and ensure their safety and effectiveness in clinical application [1]. A variety of traditional methods are applied to process crude herbs, such as boiling or steaming with licorice liquids, water, or rice wine; stir-frying with ginger juice or honey; and braising with rice wine.

**Figure 1:**
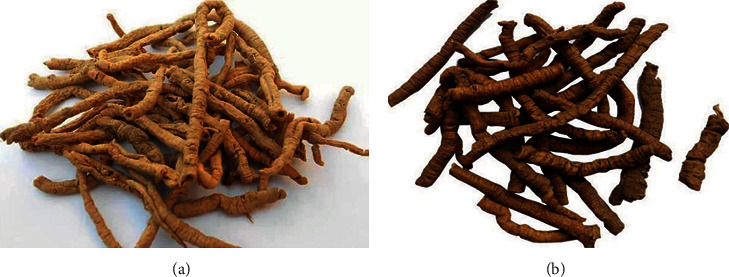
The raw *P. tenuifolia* (a) and the licorice-simmered *P. tenuifolia* (b).

*Polygala tenuifolia* Willd. (PT) (Figure 1) is a *Polygala* plant that belongs to the family Polygalaceae and is mainly distributed in China, South Korea, and the Russian Federation [2]. PT is also known as “Yuanzhi” in Chinese and has been widely used as a nootropic, anti-inflammatory, and antipsychotic medicine to cure insomnia [3], forgetfulness, neurasthenia, coughing, and soreness caused by heart and kidney disharmony [4, 5]. The main chemical components in PT include *Triterpenoid saponins*, oligosaccharide esters, xanthones, and organic acids, which have been proven to have a wide range of biological activities. *Triterpenoid saponins* are the main pharmacological substances of PT, and they exhibit tranquilizing, phlegm-expelling, and cough-stopping functions [6, 7]. Oligosaccharide esters have remarkable antidementia, antidepressant, and brain-protective activities [8]. Xanthones have antitumor, analgesic, antimycotic, and other effects [9]. Organic acids from PT exert antiseizure [10], anti-inflammatory [11], neuroprotective [12, 13], and antiamnesic effects [8].

In Chinese clinical practice, raw PT (RPT) causes some side effects, such as gastrointestinal toxicity and throat irritation, so it needs to be processed before administration. The processing methods of RPT have evolved from simple to complex, and the auxiliary materials have also been diversified. In modern times, the common processing methods of RPT include heartwood discarding, licorice boiling, licorice simmering, and honey stir-baking [14]. Among the resulting products, licorice-simmered PT (LPT) is listed in the Processing Standards of Chinese Herbal Pieces in Jiangxi Province [15]. According to the theories of TCM, licorice processing can reduce the side effects of RPT with regard to pharyngeal irritation and gastrointestinal stimulation and can promote peace of mind and intellectual development [16]. Modern pharmacological investigations have indicated that RPT can significantly inhibit gastrointestinal motility and digestive function; however, after being processed with licorice, PT has a relatively weak inhibitory effect on gastrointestinal motility and digestive function [17].

In the course of processing PT, the chemical components may be changed: the relative levels of certain components may be changed, and new components may be formed [18–21]. However, the effects of licorice simmering on the chemical components and gastrointestinal function of PT have not been reported. In this study, principal component analysis (PCA) and content determination of bioactive components were used to evaluate chemical profiles. In addition, gastrointestinal hormone levels, inflammatory cytokine levels, and histopathology were examined to compare the effects of LPT and RPT on the gastrointestinal function of rats. It is expected that the study will provide a scientific basis for the safety and effectiveness of the clinical use of PT.

## 2. Materials and Methods

### 2.1. Chemicals and Reagents

Sibiricose A5 (A5), sibiricose A6 (A6), glomeratose A (GA), onjisaponin B (OJB), and onjisaponin Z (OJZ) were supplied by Chengdu Biopurify Phytochemicals Co., Ltd. (Chengdu, China). The reference substances tenuifoliside A (TFSA), tenuifoliside B (TFSB), and tenuifoliside C (TFSC) were supplied by Shanghai Yilin Biological Technology Co., Ltd. (Shanghai, China). 3,6'-Disinapoyl sucrose (DSS) and TMCA were obtained from Chengdu Chroma Biotechnology Co., Ltd. (Chengdu, China). Tenuifoliose A (TFOA), tenuifoliose H (TFOH), and arillanin A (AA) were provided by Tianjin Shilan Biological Technology Co., Ltd. (Tianjin, China). Polygalaxanthone III (PIII) was obtained from the China National Institute for Drug and Biological Product Testing (Beijing, China). Senegenin was obtained from Chengdu Push Biological Technology Co., Ltd. (Chengdu, China). The purity of each reference standard was greater than 98%. The structures (shown in Figure 1) were fully explicated by spectral data according to the methods of Liu et al. [21].

HPLC-grade water was obtained using a Milli-Q water purification system (Millipore, Billerica, MA, USA). In addition, other reagents, such as acetonitrile, methanol, and phosphoric acid, were all of HPLC-grade and were supplied by Merck Co., Ltd. (Darmstadt, Germany). Gastrin (GAS), motilin (MTL), somatostatin (SS), substance P (SP), vasoactive intestinal peptide (VIP), interleukin-6 (IL-6), interleukin-8 (IL-8), and tumor necrosis factor-*α* (TNF-*α*) assay kits were purchased from Jiangsu Jingmei Biological Technology Co., Ltd. (Jiangsu, China).

### 2.2. Collection of Samples

All RPT samples (6 batches of samples) were collected by Shandong Baiweitang Chinese Herbal Pieces Co., Ltd. (Jinan, China) and were identified as the root of *P. tenuifolia* Willd. by Li Feng, a professor in the TCM Identification Department at Shandong University of Traditional Chinese Medicine. Six batches of samples were collected from Shanxi Province (lot numbers: 180603, 180716, and 180822) and Shandong (lot numbers: 180623, 180713, and 180906) and were deposited at the Herbarium of Traditional Chinese Medicine, School of Pharmacy, Shandong University of Traditional Chinese Medicine. Each batch of samples was divided into two groups: the RPT group and the LPT group.

LPT samples were prepared in the laboratory according to the Processing Standards of Chinese Herbal Pieces in Jiangxi Province (2008 edition) [15], which meant that 100 g of RPT was mixed with 6 g of licorice, and moderately warm water was added to the medicine jar. Then, the jar was moved to a nearby stove, dry bran was stacked around the jar, and the contents of the jar were simmered for 6 h. The liquid was then drained, and the licorice was removed. The LPT samples were dried under vacuum at 50°C.

### 2.3. Principal Component Analysis (PCA)

In order to analyze the differences between RPT and LPT, we used SIMCA 13.0 to perform unsupervised principal component analysis of the relative peak areas in HPLC chromatographs. The main chemical markers with the most influence on the classifications among different samples were determined with the help of PCA loadings biplot.

### 2.4. Sample Preparation

#### 2.4.1. Extract Preparation for Animals

RPT and LPT were selected from the samples collected and prepared as described in Section 2.2.

RPT samples were smashed into powder (through a 40-mesh screen). One kilogram of powder from each sample was extracted by ultrasonication three times with 70% ethanol (10-, 8-, and 8-fold volumes for 1 h, respectively). The extract solutions were filtered and combined, and then the ethanol was evaporated under reduced pressure at 60°C. The total crude extracts were freeze-dried and purified by macroporous resin. Finally, the RPT extract was obtained and accurately weighed.

The LPT extract was obtained following the same procedure above.

The RPT and LPT extracts were suspended in water when the rats were treated orally.

#### 2.4.2. Sample Preparation for HPLC

*Sample Preparation*. The extracts prepared in Section 2.4.1 were accurately weighed to 0.2 g and extracted with 25 ml of 70% methanol under reflux for 0.5 h. After cooling, the lost mass was replaced with 70% methanol.

*Standard Preparation*. A mixed standard solution of A5 (59.5 *μ*g/mL), A6 (49 *μ*g/mL), PIII (50 *μ*g/mL), GA (36.6 *μ*g/mL), TFSB (139.8 *μ*g/mL), DSS (200 *μ*g/mL), AA (25.5 *μ*g/mL), TFSA (106 *μ*g/mL), TFSC (78 *μ*g/mL), TMCA (52 *μ*g/mL), TFOA (82.5 *μ*g/mL), TFOH (98 *μ*g/mL), OJB (1.0 mg/mL), and OJZ (50 *μ*g/mL) was prepared in methanol and stored at 4°C. The solution was then diluted to five different concentrations to establish a calibration curve.

### 2.5. Chromatographic Conditions for HPLC

Chromatographic separation was performed in a Primaide 1430 HPLC system (Hitachi Instruments Co., Ltd.) equipped with a dual-gradient pump and a DAD detector. The data were collected and processed using a chromatographic data system. The samples were separated on Kromasil C_18_ column (250 mm × 4.6 mm, 5 *μ*m) with a mobile phase that consisted of acetonitrile (A) and 0.05% phosphoric acid solution (B). The optimized gradient program involved the following steps: 10–26% A at 0–20 min, 26% A at 20–30 min, 26–31% A at 30–31 min, 31% A at 31–40 min, 31–41% A at 40–41 min, and 41–46% A at 41–55 min. The detector wavelength was set at 320 nm. The column temperature was kept at 35°C, and the injection volume was 20 *μ*L.

### 2.6. Sample Preparation for Determination of Total Saponins

The extracts prepared in Section 2.4.1 were accurately weighed to 1.0 g and extracted with 10 ml of 70% ethyl alcohol under reflux for 1 h. The extraction solution was filtered and dried. After drying, the samples were dissolved with methanol in a 25 mL volumetric flask [22].

### 2.7. Determination of Total Saponins

Determination of total saponins was carried out using the ultraviolet spectrophotometry method. Seventy microliters of sample solution was pipetted into a 10 mL test tube with a glass stopper and evaporated to dryness in a water bath. Then, 0.2 mL of 5% vanillic aldehyde and 0.8 mL of glacial acetic acid were added. The mixture was transferred to a 60°C water bath for 15 min and cooled in ice water for 5 min for ultraviolet detection. The ultraviolet absorption was monitored at 575 nm in a UV1000 spectrophotometer. The senegenin reference standard was accurately weighed and dissolved in methyl alcohol to a final concentration of 0.7 mg/mL. Then, the solution was diluted to 5 different concentrations to establish the calibration curve [22].

### 2.8. Animal Experiments

#### 2.8.1. Animals and Housing

Male Sprague-Dawley rats (200 ± 20 g) were purchased from the Pengyue Experimental Animal Center (Jinan, China) (age: 6 weeks old) under the laboratory animal license SCXK (Lu) 20190003. All animals were randomly assigned to different groups and were subjected to adaptive training 1 week before treatment. The rats were housed under standard laboratory conditions (temperature 21–23°C, relative humidity 45–65%, and circadian cycle 12 h/12 h) with freely supplied food and water. All animal experiments were approved by the ethics committee of Shandong University of Traditional Chinese Medicine.

#### 2.8.2. Treatment of Animals

The rats were randomly divided into 5 groups (*n* = 10 in each group), including the *N* group (normal saline), L-RPT group (4.16 g/kg RPT), H-RPT group (8.32 g/kg RPT), L-LPT group (4.16 g/kg LPT), and H-LPT group (8.32 g/kg LPT) by oral gavage. During testing, the rats were weighed once a week to adjust the dosage and weighed again before sacrifice. After 15 days of intragastric administration, the rats were denied food for 12 h before blood collection. On the next day, all rats were anesthetized with isoflurane (gas concentration 5%). Abdominal aorta blood samples were collected and centrifuged at 3500 rpm for 15 min at 4°C. The serum samples were separated and stored at −80°C until analysis. In addition, the stomach and small intestine were quickly removed.

#### 2.8.3. Histopathological Analysis

The stomach and small intestine were fixed in 10% normal buffered formalin, embedded in paraffin, sectioned at 4 *μ*m, and stained with hematoxylin and eosin (H&E) for histological examination.

#### 2.8.4. Serum Biochemical Index Analysis

GAS, MTL, SS, SP, and VIP levels in the serum were detected with Enzyme Linked Immune Sorbent Assay (ELISA) kits according to the manufacturer's instructions.

#### 2.8.5. Analysis of Related Inflammatory Cytokines in the Gastrointestinal Tract

The frozen stomach and small intestine samples were thawed. Then, they were quickly cut into pieces and weighed. The ratio of gastrointestinal tissue to phosphate buffer saline (PBS) was 1 : 9 (W/W). The gastrointestinal homogenate, which was obtained by homogenization of the tissue in an ice bath, was centrifuged to obtain the supernatant. The levels of IL-8, IL-6, and TNF-*α* in gastrointestinal tissues were assayed with ELISA kits following the instructions.

### 2.9. Statistical Analysis

All data are expressed as the SD and were analyzed with one-way analysis of variance (ANOVA). Scheffe's test was used for comparisons between experimental groups in SPSS software 23.0. *P* < 0.05 was considered to indicate statistical significance.

## 3. Results

### 3.1. Analysis of Chemical Component Changes during Processing through PCA

**Figure 2:**
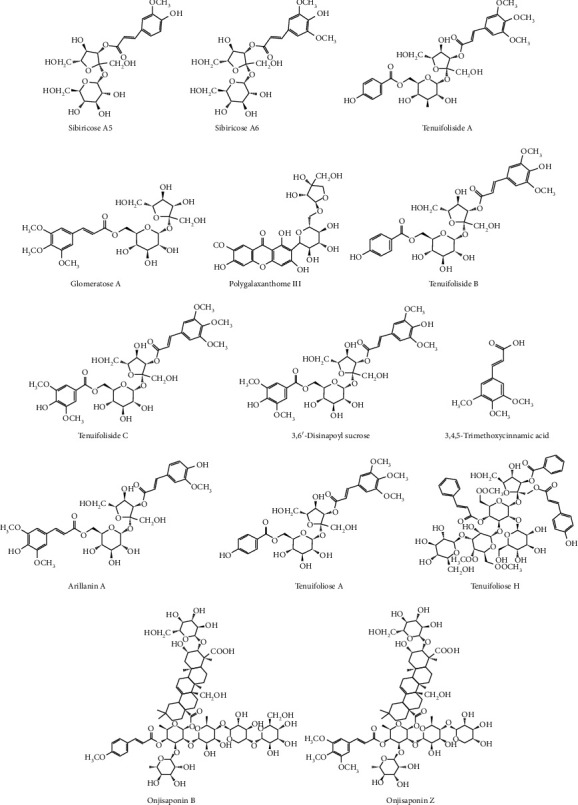
Chemical structures of 14 main active compounds of PT.

**Figure 3:**
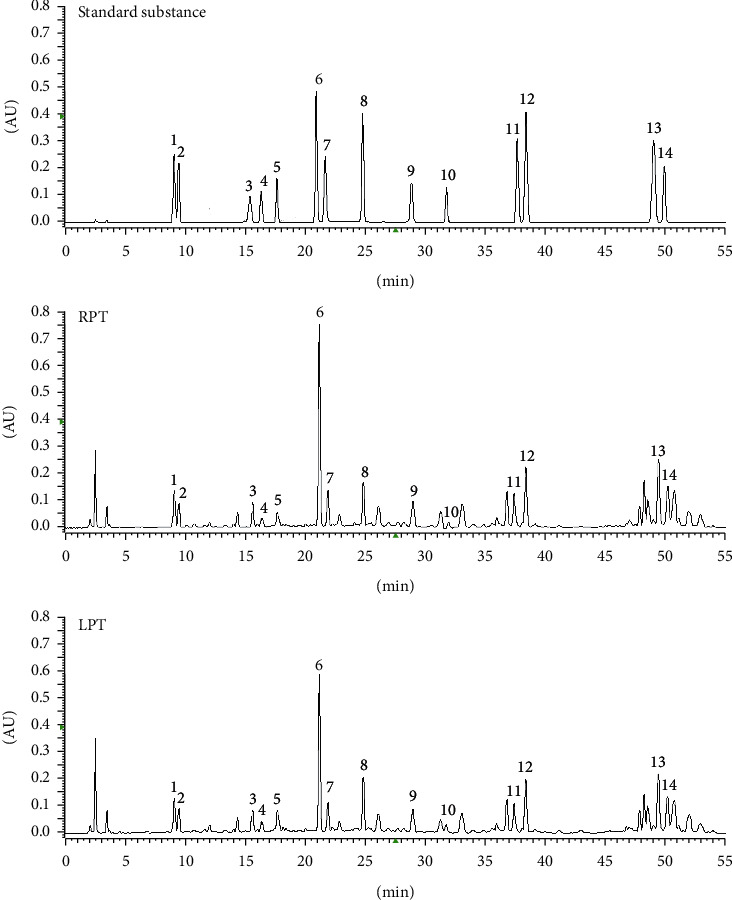
HPLC chromatogram (1: sibiricose A 5, 2: sibiricose A 6, 3: polygalaxanthones III, 4: glomeratose A, 5: tenuifoliside B, 6: 3,6'-disinapoyl sucrose, 7: arillanin A, 8: tenuifoliside A, 9: tenuifoliside C, 10: 3,4,5-trimethoxycinnamic acid, 11: tenuifoliose A, 12: tenuifoliose H, 13: onjisaponin B, 14: onjisaponin Z).

**Figure 4:**
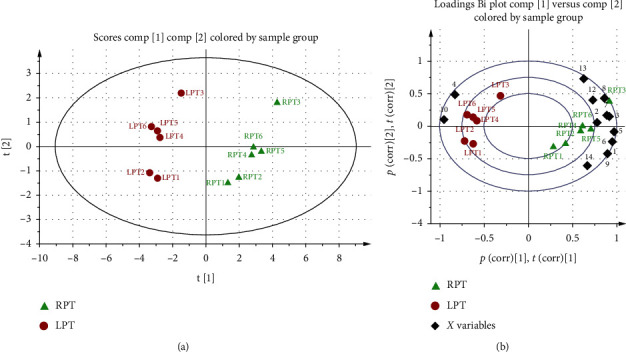
PCA score plot (a) and loading biplot (b) of RPT (green triangle) and LPT (red circle). RPT and LPT were classified into two clusters. Peaks 1, 2, 3, 5, 7, 8, 9, 11, 12, 13, and 14 are the important components to distinguish RPT and LPT, which will reduce during processing. Peaks 4 and 10 are also important components for the differences between RPT and LPT, which will increase during processing.

To analyze the PT chemical component differences before and after licorice simmering, 14 peaks were selected as characteristic peaks and identified. Their structures are shown in Figure 2. The relative peak areas were calculated for quantitative expression analysis. The HPLC chromatogram is shown in Figure 3. PCA of the relative peak areas of 14 components was performed for discrimination of the different samples. As shown in Figure 4(a), RPT and LPT occupied specific regions in the spatial distribution of principal components, which indicates that there is a significant difference between the chemical components of RPT and those of LPT. To find potential chemical markers for discrimination between RPT and LPT, extended statistical analysis was performed to generate the loadings biplot shown in Figure 4(b). Peaks 1, 2, 3, 6, 7, 8, 9, 11, 12, 13, and 14 decreased after processing, while peaks 4 and 10 increased after processing, and all of these peaks represented the most important components for distinguishing RPT from LPT.

### 3.2. Changes in Chemical Component Levels after Processing

The levels of 14 components and total saponins in RPT and LPT samples are listed in Table 1. The corresponding loadings biplot analysis was used to evaluate which chemical components changed considerably. The results showed that the levels of 14 compounds were different between LPT and RPT. Licorice processing slightly changed the levels of A5, A6, TFSA, AA, and TFSC, while it observably decreased the levels of TFSB, DSS, PIII, TFOA, TFOH, OJB, OJZ, and total saponins and markedly increased the levels of GA and TMCA.

### 3.3. Effects on Rat Gastrointestinal Tissue Histopathology

#### 3.3.1. Histopathological Examination of Stomach Tissue

**Figure 5:**
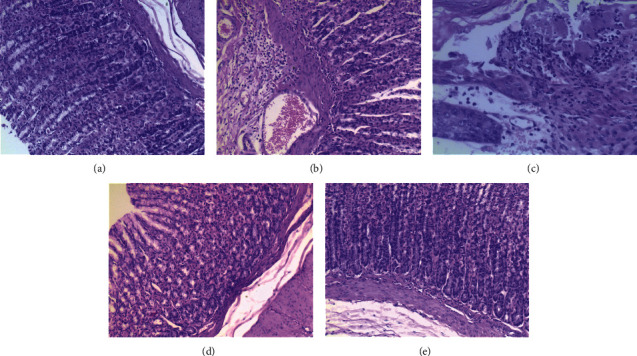
Histological observation of stomach from experimental rats was examined using H&E staining and microscopy (100× magnification). *N* group (a); L-RPT group (b); H-RPT group (c); L-LPT group (d); H-LPT group (e).

The morphology of the gastric tissue is shown in Figure 5. In the *N* group (Figure 5(a)), the structure of the gastric wall was clear; the mucosa, submucosa, muscularis mucosa, and serous membrane were normal, with no inflammatory cell infiltration, erosion, or ulceration; the blood vessels were not dilated or congested; and the endothelial cells were clear and intact without swelling. In the L-RPT group (Figure 5(b)), the blood vessels of the gastric submucosa and muscularis were dilated, with little infiltration of inflammatory cells, and the mucosal epithelium was not damaged. In the H-RPT group (Figure 5(c)), erosion of the rat cardia epithelium occurred, there were a few inflammatory cells under the gastric mucosa, the blood vessels were dilated and congested, and the gastric mucosal tissue had varying degrees of congestion and edema. In the L-LPT group (Figure 5(d)), the rats were essentially normal; the structure of the gastric wall was clear; there was no inflammatory cell infiltration, erosion, or ulceration in the mucosa, submucosa, or muscularis mucosa; and the blood vessels were not dilated or congested. In the H-LPT group (Figure 5(e)), only a few inflammatory cells infiltrated the mucosa.

#### 3.3.2. Histopathology of the Small Intestine

**Figure 6:**
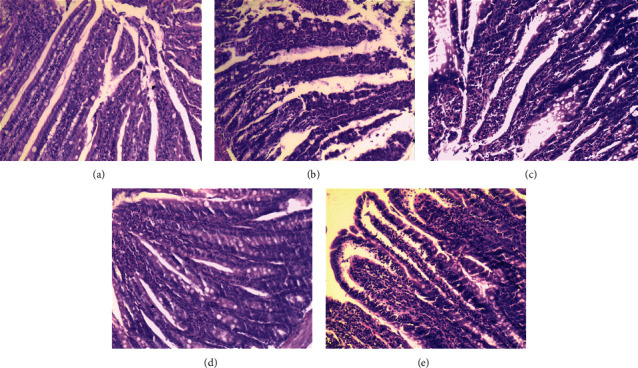
Histological observation of small intestine from experimental rats was examined using H&E staining and microscopy (100× magnification). *N* group (a); L-RPT group (b); H-RPT group (c); L-LPT group (d); H-LPT group (e).

The histology of the small intestine in the *N* group was normal (Figure 6(a)). The intestinal mucosa was intact. The muscularis mucosa and submucosa were readily identified. Inflammatory cells were occasionally seen in the villi of the small intestine mucosa, no erosion or ulceration was observed, blood vessels in villi were slightly congested, and no obvious abnormalities were found in the submucosa or muscularis mucosa. In the L-RPT group (Figure 6(b)) and the H-RPT group (Figure 6(c)), the structure was destroyed, a large number of inflammatory cells were present in the villi of the small intestinal mucosa, some sections showed lymphoid tissue hyperplasia, the mucosal epithelium of the villi was exfoliated and necrotic, and no inflammatory cell infiltration was found in the submucosa or muscularis mucosa. In the L-LPT group (Figure 6(d)), the structures of the small intestinal villi were intact, only a small number of inflammatory cells infiltrated the villi of the small intestinal mucosa, there was no obvious vasodilation, and there was no inflammatory cell infiltration in the submucosa or muscularis mucosa. In the H-LPT group (Figure 6(e)), the structures of the small intestinal villi were intact, inflammatory cell infiltration in the villi of the small intestinal mucosa was not obvious, vascular dilatation was not obvious, and no inflammatory cell infiltration was found in the submucosa or muscularis mucosa.

Examination of pathological sections of gastrointestinal tissue showed that the treatments given to the L-RPT group and the H-RPT group caused serious damage to the gastrointestinal tissues of healthy Sprague-Dawley rats. Compared with the RPT groups, the LPT groups did not exhibit obvious damage in gastrointestinal tissue.

### 3.4. Influences on Serum Biochemical Indexes

Gastrointestinal hormones are bioactive substances existing in the gastrointestinal tract that can directly or indirectly participate in gastrointestinal motility. They include mainly GAS, MTL, SP, VIP, SS, etc. Among them, GAS, MTL, and SP can promote gastrointestinal peristalsis and stimulate gastrointestinal motility, while VIP and SS can inhibit gastrointestinal motility.

**Figure 7:**
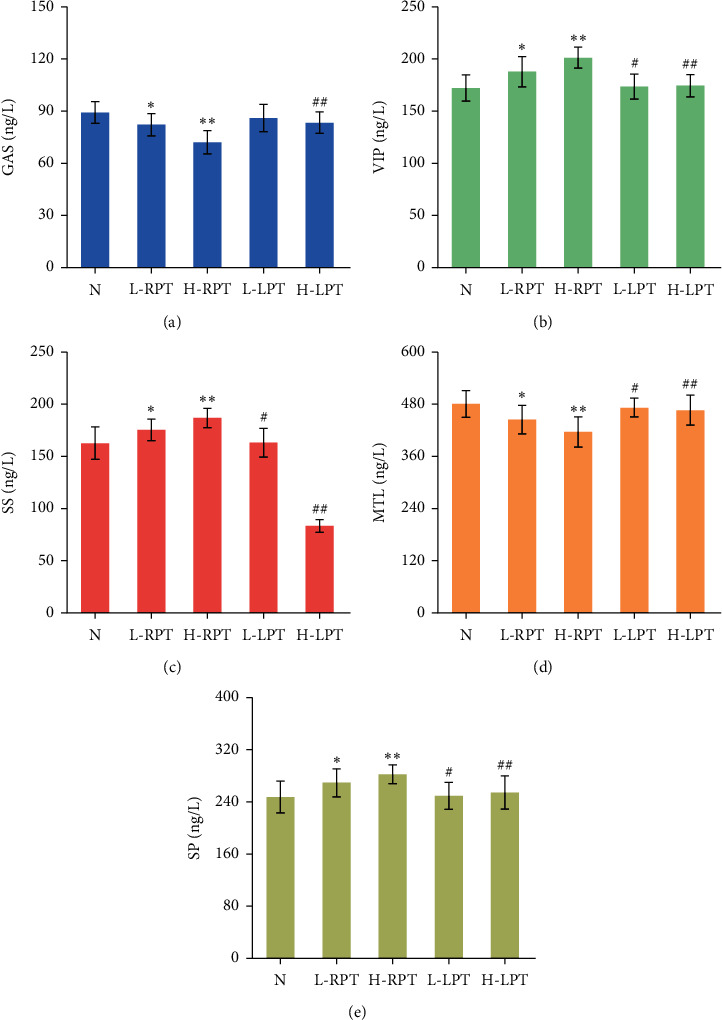
The results of serum biochemical indexes in each oral administration group of rats. Significant differences with *N* group were designated as ^*∗*^*P* < 0.05, ^*∗∗*^*P* < 0.01. Significant differences with same-dose group were designated as ^#^*P* < 0.05, ^##^*P* < 0.01.

As shown in Figure 7 and Table 2, the levels of GAS, MTL, and SP in each oral administration group were decreased to different degrees, and the levels of VIP and SS were increased to different degrees. Compared with those in the *N* group, the levels of GAS, MTL, and SP in the L-RPT and H-RPT groups were significantly lower (*P* < 0.05), while the levels of VIP and SS were markedly higher (*P* < 0.05). However, the rats in the LPT groups had no obvious differences in the above indexes. Compared with the groups treated with the same doses of RPT, the LPT groups exhibited significant differences (*P* < 0.05 or *P* < 0.01).

### 3.5. Effects on the Levels of Related Inflammatory Factors in the Gastrointestinal Tracts of Rats

**Figure 8:**
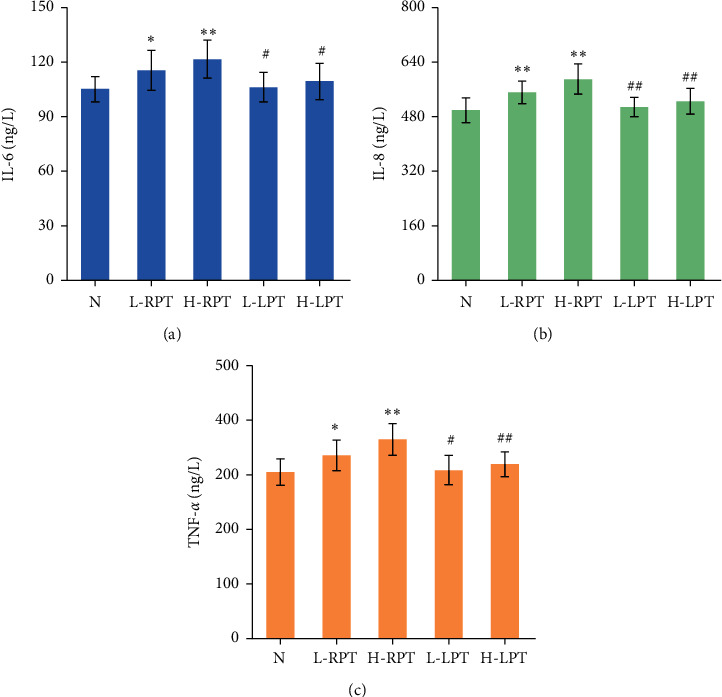
Effects of RPT and LPT on IL-6, IL-8, and TNF-*α* in stomach. Significant differences with N group were designated as ^*∗*^*P* < 0.05 and ^*∗∗*^*P* < 0.01. Significant differences with same-dose group were designated as ^#^*P* < 0.05 and ^##^*P* < 0.01.

**Figure 9:**
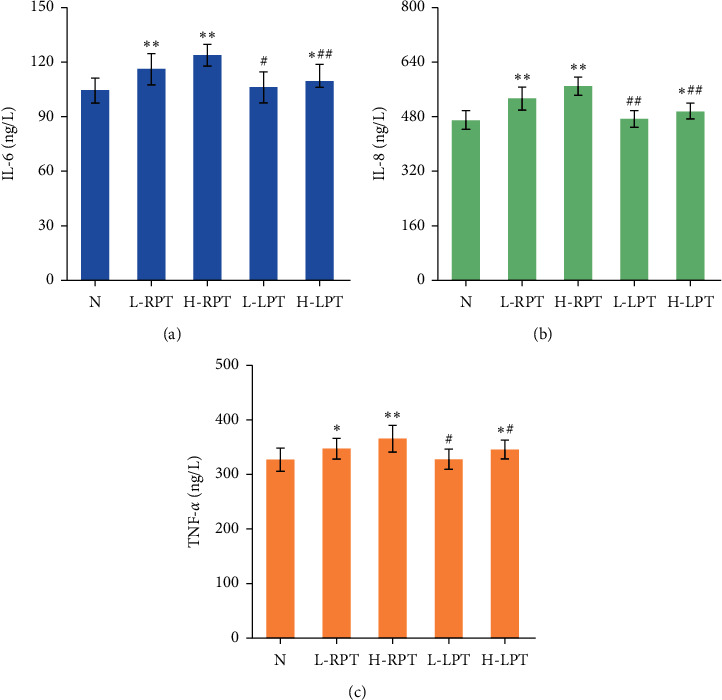
Effects of RPT and LPT on IL-6, IL-8, and TNF-*α* in small intestine. Significant differences with *N* group were designated as ^∗∗^*P* < 0.01. Significant differences with same-dose group were designated as ^#^*P* < 0.05 and ^##^*P* < 0.01.

TNF-*α* is a proinflammatory cytokine produced by specific cells under inflammatory stimulation. IL-6 and IL-8 can participate in the inflammatory response and whole-body immune response and are important indicators of the tissue inflammatory response. As shown in Figure 8, Table 3, Figure 9, and Table 4, the IL-6, IL-8, and TNF-*α* concentrations in the L-RPT and H-RPT groups were higher than those in the *N* group and the same-dose LPT groups. The levels in the H-LPT group were significantly different in the intestine (*P* < 0.05 or *P* < 0.01).

## 4. Discussion

Changes in the physical and chemical properties of TCMs result in different degrees of changes in the quality and quantity of their components. Oligosaccharide esters, saponins, and xanthones are the main chemical constituents of PT. Oligosaccharide esters are ester compounds formed by the reaction of monosaccharides, sucroses, or oligosaccharides with various phenolic acids (such as TMCA, sinapic acid, and ferulic acid). Previous studies have shown that oligosaccharide esters have unstable properties; when water is added and heat is applied, the ester bonds in their molecular structures are hydrolyzed to produce secondary glycosides and/or aglycones. OJB undergoes structural rearrangement and transformation into isomers [23–25]. The results of this study indicated that the levels of TFSB, DSS, PIII, TFOA, TFOH, OJB, OJZ, and total saponins observably decreased, while the levels of GA and TMCA markedly increased, after licorice simmering. These results were due to the hydrolysis of oligosaccharide esters and saponins during the simmering process.

Gut hormones and brain-gut peptides connect the gastrointestinal tract, brain, and gut bacteria and directly or indirectly participate in the regulation of gastrointestinal function [26, 27]. The dynamic balance of various gut hormones and brain-gut peptides is crucial to the normal functioning of the gastrointestinal tract. It is known that GAS and MTL can promote gastric acid and pepsin secretion, stimulate gastrointestinal peristalsis, and promote gastric emptying [28]. In contrast, VIP, SS, and SP are important inhibitory neurotransmitters in the intestine that can inhibit digestive juice secretion and gastrointestinal emptying [26, 29]. In this study, there were significant differences in the gastrointestinal function-related proteins GAS, MTL, VIP, SS, and SP between the RPT groups and the *N* group, which suggested that gastrointestinal injury occurred in the RPT groups. Compared with the same-dose RPT groups, the LPT groups had significant differences in the levels of these gastrointestinal hormone indicators. This suggests that the inhibitory effect of PT on gastrointestinal function in rats may be related to gastrointestinal hormone disruption. Simmering with licorice reduced the inhibitory effect of RPT on the gastrointestinal function of rats.

TNF-*α* is an important factor for biological immune defense and maintenance of internal environment stability, and an increase in the TNF-*α* level is related to gastrointestinal tissue injury [30]. IL-6 is a chemokine that can destroy the gastric mucosal barrier, thereby aggravating gastric mucosal inflammatory injury [31]. IL-8 is a key mediator associated with inflammation and plays a role as an autocrine growth factor in intestinal diseases [32]. Upregulation of proinflammatory cytokine expression is also positively correlated with the severity of inflammation. The expression of IL-6, IL-8, and TNF-*α* in the stomachs and small intestines of Sprague-Dawley rats was detected in our study, and we found that the levels of IL-6, IL-8, and TNF-*α* in gastrointestinal tissue were markedly increased in the L-RPT, H-RPT, and H-LPT groups, showing a certain inflammatory effect of the treatments; however, the inflammatory effect of L-LPT was relatively weak. These results indicate that PT can cause gastrointestinal inflammation and gastrointestinal mucosal damage in rats and that the above effects are obviously weakened after licorice simmering. The gastrointestinal irritation caused by PT in rats is related to the release of gastroenteritic factors.

Licorice is a common detoxifying Chinese medicine that achieves effects such as tonification of the spleen and Qi, heat clearance and detoxification, and pain relief [33]. The active ingredients in licorice, flavonoids, possess anti-inflammatory, antioxidative, antitumoral, and analgesic effects [34]. Licorice flavonoids can effectively inhibit gastric mucosal injury, promote gastric juice secretion, increase GAS levels and pepsin activity to enhance gastric mucosal defense, inhibit inflammatory factor release, and reduce mucosal inflammation [35–37]. According to other studies, total saponins and OJB are irritating to the gastrointestinal tracts of rats, and the toxicity of PT is proportional to the content of total saponins. The higher the content of total saponins, the greater the toxicity [38, 39]. The results of this study showed that the levels of total saponins and OJB were decreased significantly after simmering. In addition, after licorice simmering, the inhibitory effect on gastrointestinal function was weakened, and the damage caused by RPT to the gastrointestinal mucosa was reduced. The findings are related to the transformation of saponins and the addition of licorice. In the future, we will further study the mechanism by which the gastrointestinal toxicity of RPT was reduced. This study can provide a scientific basis for the rational clinical use of processed PT and provide a reference for research on the principles of LPT processing.

## 5. Conclusions

In summary, the levels of chemical compounds were changed after processing. The pharmacological experimental results demonstrated that processing of RPT significantly alleviated its inhibitory effect on gastrointestinal function in rats. Licorice simmering reduced stimulation of the gastrointestinal tract by regulating the levels of serum GAS, MTL, SS, SP, and VIP; improving the gastrointestinal inflammatory response; and restoring the levels of TNF-*α*, IL-6, and IL-8. Changes in chemical composition form the internal material basis for the changes in the medicinal properties and gastrointestinal effects of PT after processing. This study might provide a theoretical basis for the clinical application and further development and utilization of PT. The next step is to study the mechanism of PT detoxification with a cell or animal model and search for the related inflammatory signaling pathway through protein and gene expression analysis.

## Figures and Tables

**Table 1:** The contents of 15 compounds and total saponins in RPT and LPT (%, mean ± SD, *n* = 6).

Sample	A 5	A 6	PIII	GA	TFSB	DSS	AA	TFSA	TFSC	TMCA	TFOA	TFOH	OJB	OJZ	Total saponins
RPT	0.173 ± 0.011	0.123 ± 0.007	0.356 ± 0.020	0.101 ± 0.006	0.464 ± 0.043	1.377 ± 0.237	0.048 ± 0.004	0.742 ± 0.137	0.318 ± 0.021	0.096 ± 0.017	0.145 ± 0.015	0.480 ± 0.059	2.104 ± 0.315	0.540 ± 0.013	3.373 ± 0.162
LPT	0.163 ± 0.007	0.115 ± 0.007	0.321 ± 0.022^*∗*^	0.125 ± 0.005^*∗*^	0.391 ± 0.068^*∗*^	1.081 ± 0.136^*∗*^	0.043 ± 0.003	0.555 ± 0.065	0.290 ± 0.023	0.124 ± 0.0185^*∗*^	0.121 ± 0.013^*∗*^	0.410 ± 0.047^*∗*^	1.924 ± 0.310^*∗*^	0.489 ± 0.034^*∗*^	2.894 ± 0.219^*∗*^

Significant differences with RPT were designed as ^*∗*^*P* < 0.05.

**Table 2:** Serum biochemical indexes in each oral administration group (mean ± SD, *n* = 10).

Group	GAS (ng/L)	VIP (ng/L)	SS (ng/L)	MTL (ng/L)	SP (ng/L)
*N*	89.17 ± 6.24	172.43 ± 12.62	162.63 ± 15.62	481.12 ± 30.82	247.56 ± 24.46
L-RPT	82.27 ± 6.45^*∗*^	188.13 ± 14.46^*∗*^	175.55 ± 10.45^*∗*^	444.57 ± 32.66^*∗*^	269.44 ± 21.45^*∗*^
H-RPT	72.11 ± 6.77^∗∗^	201.63 ± 10.16^∗∗^	186.91 ± 9.32^∗∗^	416.28 ± 34.72^∗∗^	282.65 ± 14.27^∗∗^
L-LPT	86.03 ± 7.82	173.82 ± 11.91^#^	163.18 ± 13.64^#^	472.31 ± 21.23^#^	249.51 ± 20.66^#^
H-LPT	83.39 ± 6.18^##^	174.62 ± 10.85 ^##^	165.86 ± 11.82^##^	466.48 ± 34.21^##^	254.62 ± 25.53^##^

Significant differences with *N* group were designated as ^*∗*^*P* < 0.05 and ^*∗∗*^*P* < 0.01. Significant differences with same-dose group were designated as ^#^*P* < 0.05 and ^##^*P* < 0.01.

**Table 3:** Effects of RPT and LPT on IL-6, IL-8, and TNF-*α* in stomach (mean ± SD, *n* = 10).

Group	IL-6 (ng/L)	IL-8 (ng/L)	TNF-*α* (ng/L)
*N*	105.13 ± 6.95	499.43 ± 36.44	305.05 ± 24.15
L-RPT	115.52 ± 10.92^*∗*^	551.73 ± 32.94^∗∗^	335.88 ± 28.19^*∗*^
H-RPT	121.60 ± 10.40^∗∗^	590.87 ± 44.22^∗∗^	365.00 ± 28.85^∗∗^
L-LPT	106.22 ± 8.11^#^	508.59 ± 28.08^##^	308.70 ± 26.98^#^
H-LPT	109.40 ± 9.95 ^#^	526.84 ± 38.42^##^	319.32 ± 22.67^##^

The values are presented as mean ± SD. Significant differences with *N* group were designated as ^*∗*^*P* < 0.05, ^∗∗^*P* < 0.01. Significant differences with same-dose group were designated as ^#^*P* < 0.05 and ^##^*P* < 0.01.

**Table 4:** Effects of RPT and LPT on IL-6, IL-8, and TNF-*α* in small intestine (mean ± SD, *n* = 10).

Group	IL-6 (ng/L)	IL-8 (ng/L)	TNF-*α* (ng/L)
*N*	104.43 ± 6.82	469.66 ± 27.30	326.99 ± 21.23
L-RPT	116.13 ± 8.54^∗∗^	532.93 ± 33.63^∗∗^	347.13 ± 19.09^*∗*^
H-RPT	123.70 ± 5.94^∗∗^	569.12 ± 26.57^∗∗^	365.53 ± 24.39^∗∗^
L-LPT	106.23 ± 8.55^#^	472.83 ± 24.48^##^	327.85 ± 18.42^#^
H-LPT	112.46 ± 6.18^∗##^	495.20 ± 23.07^∗##^	345.45 ± 17.43^∗#^

Significant differences with *N* group were designated as ^*∗∗*^*P* < 0.01. Significant differences with same-dose group were designated as ^#^*P* < 0.05 and ^##^*P* < 0.01.

## Data Availability

All data generated or analyzed during this study are included in this paper. The data used to support the findings of this study available from the corresponding author upon request.
